# Risky Treatments: A Jewish Medical Ethics Perspective

**DOI:** 10.5041/RMMJ.10217

**Published:** 2015-07-30

**Authors:** Avraham Steinberg

**Affiliations:** Director, Medical Ethics Unit, Shaare Zedek Medical Center, Jerusalem, Israel

**Keywords:** Autonomy, Jewish medical ethics, medical ethics, risky treatments

## Abstract

The Jewish principle concerning a decision with regard to a dangerous treatment is as following: A patient who is estimated to die within 12 months because of a fatal illness is permitted to undergo a treatment that on the one hand may extend his life beyond 12 months, but on the other hand may hasten his death. There are, however, several limitations to this ruling related to the chances of success with the proposed treatment, the nature of the treatment, whether it is intended to be curative or merely to postpone the danger and death, whether the treatment is absolutely necessary, and others. One is not obligated to undergo a dangerous treatment, but one is permitted to do so. The permissibility to forfeit a short life expectancy in order to achieve more prolonged life applies only with the patient’s consent. That consent is valid and is not considered a form of attempted suicide. Neither is a refusal to submit to treatment considered an act of suicide; the patient has the right to refuse a dangerous procedure. In all situations where a permissive ruling is granted for a patient to endanger his short life expectancy, the ruling should be arrived at after careful reflection and with the approval of the rabbinic authorities acting on the recommendation of the most expert physicians.

## JEWISH ETHICAL PRINCIPLES

The basis, validity, and source of Jewish ethics are rooted in the belief in God and His *Torah* (Bible), whereas the basis of secular ethics is based primarily upon humanistic and rational intellect.

The following are some basic principles of Jewish ethics as viewed by Orthodox Judaism:

- In Judaism, there is no basic difference between laws/regulations (*Halakhah*) and morals/ethics because both are integral parts of the *Torah* and their validity flows from the power of the *Torah* and the Divine revelation. Therefore, basic principles, discussions, and debates on Jewish *ethical* issues do not differ from those of Jewish *legal* issues.
- Jewish ethics includes guidelines for proper conduct of man in relation to his fellow man as well as in man’s relation to God. Therefore, there is no difference in the binding nature of the law between the prohibitions of stealing, killing, falsehood, revenge, carrying a grudge, and the like, and the laws prohibiting idol worship, Sabbath desecration, eating on *Yom Kippur*, and the like. Nor is there a difference between the obligations of giving charity, visiting the sick, burying the dead, caring for orphans and widows and their like, and the observance of dietary laws, eating unleavened bread (*matzah*) on Passover, sitting in the *Sukkah* on Tabernacles (the holiday of *Sukkot*), and the like.
- According to the *Torah* and Jewish law (*Halakhah*) one is obligated not only to refrain from doing bad, but one must do good by being compassionate and charitable with one’s fellow human beings as it is written, “turn from evil and do good.”^1^ These are two equal parts of the Jewish ethical obligation. Therefore, not only are harmful acts such as stealing, wounding, and killing prohibited, but there are positive commandments: to give charity, to visit the sick, to be hospitable, to return lost objects, and the like.
- Jewish legal-moral principles require not only proper acts but also proper thoughts and intentions. The *Torah* forbids hatred, covetousness, revenge, carrying a grudge, and the like, and requires one to love God, to love one’s fellow man, and to love a stranger, and the like, in spite of the obvious difficulties in controlling one’s thoughts.

The Bible and Talmud are replete with references to proper conduct, both between man and man and man and God.

Jewish ethical teaching involves general concepts and principles on the one hand, and specific rules and regulations on the other. The Bible cites a number of basic principles about the proper relationship between man and man, such as:

- “Love your fellow man as yourself”^2^—this is a major principle in the *Torah*^3^,^4^; “what is hateful to you, do not do to your neighbor, that is the whole *Torah*, while the rest is commentary, go and learn it.”^5^
- “Do not profane the name of your God,”^6^ namely do not conduct yourself in a way that profanes the name of God.^7^,^8^
- “You shall do what is righteous and good in the eyes of the Lord.”^9^
- “Observe Justice and perform righteousness.”^10^
- “Despise evil and love good, and establish justice by the gate.”^11^
- “Do justice, love kindness, and walk humbly with your God.”^12^
- “Thou shall not stand aside while your fellow’s blood is shed.”^13^
- “Righteousness, righteousness shall you pursue.”^14^
- “That you may walk in the way of the good, and keep the paths of the righteous.”^15^
- “Its ways are ways of pleasantness, and all its pathways are peace”^16^ etc.

However, the Jewish ethical system, like the *halakhic* system, is not satisfied with general theoretical rules alone but is filled with practical and individual guidelines. The *Torah* requires every human being to strive for perfection in one’s conduct vis-à-vis another person, in actions, in speech, and in thought, and not just abstract general good behavior (see the portrayal of righteous and proper behavior in the Bible,^17^–^20^ and others).

## JEWISH MEDICAL ETHICS PRINCIPLES

- There is an obligation upon the physician to heal the sick. The role of a physician is not optional in Jewish law but rather obligatory.
- There is an obligation upon the patient to seek medical help. Whenever a treatment for an illness is assumed to be medically beneficial there is an obligation upon a patient to undergo such treatment. He who refrains from doing so is described in Scripture: “And surely your blood of your lives will I require.”^21^
- There is an obligation of respect and dignity toward fellow man.
- There is a call for solidarity and mutually shared values and duties in society rather than individualism and extreme autonomy.

## JEWISH MEDICAL ETHICS VERSUS SECULAR MEDICAL ETHICS

Jewish medical ethics, in terms of the application of *halakhic* (Jewish legal) and Jewish ethical principles to the solution of health-related problems, differs from secular medical ethics on four planes: (1) the range of discussions and attitudes; (2) the methods of analysis and discussion; (3) the final conclusions; and (4) the basic principles.^22^,^23^

### The Range of Discussions and Attitudes

*Halakhah* addresses all the medical ethical questions which secular medical ethics raises, whether old or new. *Halakhah* also addresses specific medical issues that affect only Jews who observe the precepts of the *Torah*. The basic Jewish approach is the same for questions relating to the terminally ill, abortion, organ transplantation, and questions relating to the treatment of patients on the Sabbath, the laws of seclusion, or the laws of a menstruating woman.

### Methods of Analysis and Discussion

Jewish medical ethics analyzes medical ethical questions with the same methods and *halakhic* principles used for any *halakhic* analysis using basic principles and sources enunciated in the Talmud, Codes of Jewish law, and the Responsa literature of all generations. The scientific or medical data are presented, and the relevant *halakhic* sources are then applied to the data. It is not always easy to arrive at a *halakhic* conclusion regarding a medical question. A far-reaching knowledge of *Halakhah* as well as an expert and precise understanding of the relevant scientific facts is required in order to arrive at the proper *halakhic* conclusion.

Judaism in general prefers the casuistic approach to resolve *halakhic* questions. This means that one must examine each situation according to the individual circumstances and develop the response according to the specific details, nuances, and characteristics of that situation, using many of the basic *halakhic* rules, regulations, and principles. This is the methodology of the rabbinic Responsa literature and is ideally suited for medical questions where the circumstances differ from patient to patient. By contrast, the current approach of Western secular medical ethics uses a limited number of ethical principles and applies them to all situations involving medical ethical questions.

The *halakhic* construct in resolving a medical ethical question is a tripartite one involving the patient and/or family, the physician and the medical team, and the rabbinic decisor. The patient is obligated to seek the best possible medical care. He has the autonomous right to choose his physician and his rabbinic decisor and has the right to make his personal wishes known. The physician is obligated to treat the patient and must offer the best diagnostic and therapeutic interventions according to his knowledge and judgment. The rabbinic decisor is obligated to understand all the facts of the medical questions, to consider the views presented by the patient and the physician, and then to decide according to *halakhic* principles and precedencies how to proceed in any given situation. His decision is binding both for the patient and for the physician. It is obvious that this construct applies only to medical situations which have *halakhic* ramifications. Pure medical decisions are decided upon by the physician and the patient. This construct can be termed a religious-paternalistic approach, which restricts the patient’s as well as the physician’s autonomy and requires acceptance of the *halakhic* decision, but it negates personal paternalism.

### Final Conclusions

*Halakhah* attempts to give final and operative decisions to questions posed to the rabbinic decisor. Since Judaism is not just an academic discipline, the goal of studying and teaching Jewish medical ethics, as in all other areas of *Torah* learning, is to put *Torah* law and ethics into practice. This is in contrast to secular medical ethics, which views its function as defining the relevant ethical dilemmas, sharpening the focus of the various views, but not necessarily arriving at final and practical conclusions.

Since time immemorial, however, Rabbis have differed in their opinions, and not always is the final decision unanimous. This situation is no different than any other normative legal matter. Mechanisms exist in *Halakhah* to decide among the various opinions. In this respect, there is no difference between a medical question and any other question in any area of Judaic practice or belief.

### Basic Principles

It is important to delineate the basic principles of Jewish medical ethics as compared to secular medical ethics.

Jewish ethics, including Jewish medical ethics, is based upon duties, obligations, commandments, and reciprocal responsibility. The word “right” in its modern sense, meaning “I am entitled to it,” does not exist in biblical or talmudic literature. By contrast, secular medical ethics is based heavily on the concept of rights and autonomy. This approach justifies human decisions that cannot be criticized as long as they do no harm to others. Judaism, however, requires self-fulfillment based on obligatory and binding moral requirements that are beyond the personal, temporal feeling of individuals but rather founded on values mutually beneficial to society.

Judaism recognizes absolutism only with respect to the Divine source of authority of Jewish law, the supreme authority of the prophets who speak the words of God, and the eternity of *Torah*. Judaism does not, in general, subscribe to a set of principles and values as absolute imperative categories but rather favors a middle-of-the-road approach, the “path of the golden mean,” which is a proper balance between different values or laws in any specific case, as stated by Solomon,^24^ by Maimonides,^25^,^26^ and by Rabbi Abraham de Boton.^27^ The ethical imperative for the average person is to conduct oneself properly with the appropriate balance between opposing values and to avoid extreme positions. Hence, for Judaism there is no definitive value that is absolute, such that takes precedence in every case or situation. Various values have different moral weight, and there is a system for ascribing priorities in specific situations where conflicting values exist. This view is based on the principle that “the *Torah* was not given to ministering angels”^28^ but to ordinary human beings who, by definition, are not perfect.

The physician–patient relationship in Judaism is not a voluntary-contractual arrangement but a Divine commandment and obligation on both sides. The patient is commanded to seek healing from the physician and to prevent illness if possible. The physician is obligated to heal and is considered to be the messenger of God in the care of patients. The patient is not free to decide autonomously to refuse treatment, which might be beneficial or save his life. He is prohibited from relying on miracles, but must do whatever is necessary to heal himself according to standard medical practice.

In Judaism, the value of human life is supreme; therefore, to save a life nearly all biblical laws are waived. This approach is in contrast to the secular ethical view that considers human life to be one of many values and often gives greater weight to “the quality of life.” Nonetheless, even in Judaism, the value of human life is not absolute, and in certain rare and well-defined circumstances other values may supersede it. This, however, does not in any way diminish the supreme value of human life in Judaism.

The four basic principles widely accepted in secular medical ethics nowadays are also accepted as important values in Judaism, but they do not receive the same weight in the Jewish tradition.

The principle of autonomy which is dominant in Western secular medical ethics is modified in Judaism. Judaism asserts that man was created in the image of God^29^ and that all people are, therefore, considered special and equal.^30^–^32^ Thus, Judaism requires that people must respect and help one another. Judaism also accepts a degree of patient autonomy in the physician–patient relationship. However, in certain situations in which autonomy conflicts with other fundamental principles of Judaism, such as the obligations to preserve one’s health and life, to avoid harming others, and to do good for others, *Halakhah* may be in direct conflict with autonomy.

In Judaism, man is said to have free will and choice. This does not mean that he is permitted to choose to live immorally or to violate *Torah* laws. A person is commanded to live within *halakhic* norms, and thus his autonomy and free choice are restricted. Decision-making in areas which do not involve *Halakhah* can be totally autonomous. However, in every life situation in which there is a clear *halakhic* position any observant Jew, be he the physician or the patient, must always act within the parameters of *Halakhah* and not on one’s own inclinations and desires.

The principles of beneficence and non-maleficence are clearly defined axioms in Judaism which prohibit the intentional harming of another person either physically, emotionally, or financially, or by defamation or by an attack on objects owned by others. In addition, Jewish law clearly requires not only the avoidance of harm to others but the active doing of good to others. Sometimes, punishment is inflicted for not doing so. This approach is in contradistinction to secular law and ethics which usually only require one to avoid harm to others but do not obligate one to do good for others. Acts of kindness are considered praiseworthy but not specifically required in secular law and ethics as they are in Jewish law. Thus, coming to the aid of a stranger (“good Samaritanism”), considered a supererogatory act in most Western societies, is obligatory in Judaism.

## RISKY TREATMENTS

### Early Sources

The main source dealing with this question is the biblical story of the four lepers who sat at Jerusalem’s gates during the war between Israel and Aram:

And they said one to another: “why sit here until we die? If we say: we will enter the city when the famine is in the city we shall die there; but if we remain here, we die also; therefore, let us fall into the camp of Aram; if they permit us to live, we shall live, and if they kill us, we shall die.”^33^,^34^

The Talmud concludes from this episode that one may forfeit short time survival (*chayei shaah*) if there is any hope for long life (*chayei olam*).^35^,^36^

Another Talmudic source seems to contradict this rule. It is stated: one should desecrate the Sabbath by removing debris from a collapsed house in order to save a life of the hour (temporary life).^37^ This denotes the concept that even a very short span of life takes precedence over one of the strictest laws in Judaism, namely desecration of the Sabbath.

The answer to this contradiction is given by commentaries of the Talmud: In both instances we do whatever is good for the patient with a life of the hour. Hence, in the case of desecrating the Sabbath—in order to give the person a chance to survive we need to act, because if one does not interfere, the patient will certainly die; in the case of treating a terminally ill patient—we need to act in order to give the patient a chance to survive, because if one does not take the chance of treatment, he surely will die.^38^

### Jewish Ethical Rulings

The Jewish principle concerning a decision of a dangerous treatment is as follows: A patient who is estimated to die within 12 months because of a fatal illness (this defines “life of the hour”) is permitted to undergo a treatment that on the one hand may extend his life beyond 12 months, but on the other hand may hasten his death (shorter than the natural course of his lethal illness).^39^–^45^

There are, however, several limitations to this ruling:

- Some Rabbis limit this permissive ruling to situations where the chances of success with the proposed treatment are at least 50%.^46^ Other Rabbis rule that only if the chances of mortality by the proposed risky procedure is less than 30% is it permissible to undergo the treatment.^47^ Yet other Rabbis rule that as long as there are any chances for prolonging life it is permissible, because it is being done for the patient’s benefit with the chance, even remote, of prolonging the patient’s life.^48^,^49^
- Some Rabbis limit this permissive ruling to situations where the treatment’s intent is curative; however, if the treatment will not eliminate the illness or the danger but will merely postpone the danger and death, it is prohibited if the treatment itself may actually hasten the patient’s death.^49^
- One is not obligated to undergo a dangerous treatment, but one is permitted to do so. However, if the chances of success are very high, one is obligated to submit to potentially life-saving treatment.^49^
- The permissibility to endanger oneself in order to achieve a cure from an illness applies if the treatment or surgery is absolutely necessary and without which the patient will die. However, if there is doubt, so that the patient might survive without the treatment, and the treatment itself might hasten death, it is prohibited to endanger oneself.^50^
- Some Rabbis write that if a patient is not in danger of dying from an illness but is suffering terribly, and a treatment can relieve the suffering but the treatment may cause the death of the patient, it is prohibited to use it.^49^ Other Rabbis rule that in such a situation, one should not instruct the patient to undergo that dangerous treatment, but if the patient requests it, it is permissible in order to alleviate his suffering.^51^
- The permissibility to forfeit a short life expectancy in order to achieve more prolonged life applies only with the patient’s consent. That consent is valid and is not considered a form of attempted suicide. Neither is a refusal to submit to treatment considered an act of suicide; the patient has the right to refuse a dangerous procedure.^49^,^51^
- Some Rabbis rule that in all situations where a significant risk exists one may proceed with the treatment if a majority of expert consultants agree, and with the approval of the rabbinical advisor as well.^52^ In all situations where a permissive ruling is granted for a patient to endanger his short life expectancy, the ruling should be arrived at after careful reflection and with the approval of the rabbinic authorities acting on the recommendation of the most expert physicians.^53^
